# Retrospective analysis on distribution and antifungal susceptibility profile of *Candida* in clinical samples: a study from Southern India

**DOI:** 10.3389/fpubh.2023.1160841

**Published:** 2023-05-12

**Authors:** Umamaheshwari S., M. N. Sumana

**Affiliations:** ^1^Department of Microbiology, School of Life Sciences, JSS Academy of Higher Education and Research, Mysuru, Karnataka, India; ^2^Department of Microbiology, JSS Medical College and Hospital, JSS Academy of Higher Education and Research, Mysuru, Karnataka, India

**Keywords:** *Candida albicans*, non-albicans *Candida*, species distribution, antifungal susceptibility, retrospective-analysis

## Abstract

**Introduction:**

*Candida* is one of the rising primary causes of infections connected with health care. However, their distribution and susceptibility patterns vary widely amongst different regions.

**Method:**

The study was carried out to retrospectively analyze the distribution of *Candida* in various clinical samples, their species types and susceptibility, in a tertiary care hospital, in India for 4 years using the Vitek-2 database.

**Results:**

*Candida* infection was identified in 751 clinical samples, and the major source of infection was found to be urine samples accounting for about 58.32%. A total of 18 different *Candida* species were recorded. Non-albicans *Candida* (NAC) 73.64% (*n* = 553) predominated *Candida albicans* 26.36% (*n* = 198). *Candida tropicalis* was found to be identified at a higher frequency followed by *C. albicans*, *Candida glabrata* and *Candida parapsilosis*. *Candida tropicalis* was the only species which were recovered from bile; *Candida pelliculosa* was recorded merely from blood and *Candida lipolytica* from urine and blood and not in any other samples. In vaginal swabs, *C. albicans* accounted for 63.64% (*n* = 14) compared to NAC 36.36% (*n* = 8). The susceptibility test revealed that 75.44% (*n* = 559) isolates were susceptible and 24.56% (*n* = 182) were resistant to one or more drugs tested. Major resistance was exhibited to flucytosine by *C. tropicalis* 77.46% (*n* = 55) compared to *C. albicans* 11.27% (*n* = 8). Apart from *C. albicans*, NAC-*C. tropicalis*, *C. glabrata and Candida krusei* showed resistance to echinocandins, and *Candida haemulonii* to amphotericin-B.

**Conclusion:**

The knowledge of the incidence, resistance and emergence of different species might guide clinicians to select an appropriate antifungal therapy and plan effective strategies to control invasive and systemic *Candida* infections.

## 1. Introduction

*Candida* has been documented as the most common fungi associated with nosocomial infections due to their invasive interventions and indwelling nature in prosthetic devices (1). Also, they are the most common opportunistic infections in patients with immunocompromised conditions and those with impaired physiological and cellular barriers (2). Their dimorphic nature, adherence to host tissues/medical devices, invasive character, biofilm formation and secretion of extracellular hydrolytic enzymes have swapped the commensal character to pathogenic. Additionally, the factors such as profuse usage of wide-spectrum antibiotics, and corticosteroids, expanded use of antifungal drugs, and geographical difference in the epidemiology have increased their occurrence rate, distribution, and diversity frequency (3–8).

Among *Candida* species, *Candida albicans* is recorded as the most prevalent species, but its prevalence varies considerably depending on geographical regions; and a shift to non-albicans *Candida* (NAC) has been observed globally (9, 10). NAC have emerged as clinically interested pathogens and are exhibiting a high degree of resistance to the azole group of antifungals compared to *C. albicans* (11–13).

The rise of superbug *Candida* infections *auris* with increased morbidity and mortality in different parts of the continent is challenging the diagnosis methods and treatments (14). *Candida* is known to cause infections in cutaneous, mucosal, and deep-seated organs. Various species have been isolated from multiple clinical sites. The increased incidence of candidiasis, the surge of NAC, and the change in susceptibility patterns have emphasized the need to monitor laboratory data and select the appropriate antifungal for therapy (14–17). The knowledge of epidemiological surveillance on species distribution and their emerging resistance to antifungal agents can be the guide for effective management and antifungal stewardship.

The study aimed to retrospectively analyse the data on *Candida* species diversity, their distribution in different clinical samples and susceptibility profile in a tertiary care hospital, in Karnataka, India. Though individual institution data does not represent the total population, surveillance on species distribution and antifungal susceptibility test (AST) of a particular geographical area improve clinical decisions.

## 2. Materials and methods

A retrospective analysis was carried out to understand the distribution of *Candida* species in different clinical samples and their drug susceptibility pattern from the Department of Microbiology database of an 1800-bed tertiary care hospital in Mysuru, Karnataka, India, for 4 years. The database included patient identifiers, hospital wards/outpatient services/departments, sample collection date, specimen type, body site of isolation and results of speciation and AST pattern.

The samples were processed as per the standard microbiological procedures. The isolates were identified to the species level, and AST patterns were determined using the Vitek 2 system (BioMerieux, France). The AST panel included the antifungals such as amphotericin B, 5 flucytosine, caspofungin, micafungin, fluconazole, and voriconazole. The susceptibility results were concordant with CLSI and EUCAST Methodologies and were interpreted as sensitive (S), intermediate (I) and resistant (R) (18, 19).

The clinical samples received by the laboratory for routine analysis were processed as per the standard protocols. Therefore, the study did not require oversight by the institutional ethics committee because of its descriptive nature.

### 2.1. Statistical analysis

Descriptive analysis was carried out to analyse the distribution of *C. albicans* and NAC, in different clinical samples and susceptibility to antifungal drugs year-wise. The Mann–Whitney *U*-test and Pearson’s Chi-square Test were used to compare the differences and find the association between variables. The Mann–Whitney *U*-test with a 5% significance level and Pearson’s Chi-square Test with a *p*-value <0.05 was significant. Statistical analysis was performed using SPSS 20.0 program.

## 3. Results

**Figure 1:**
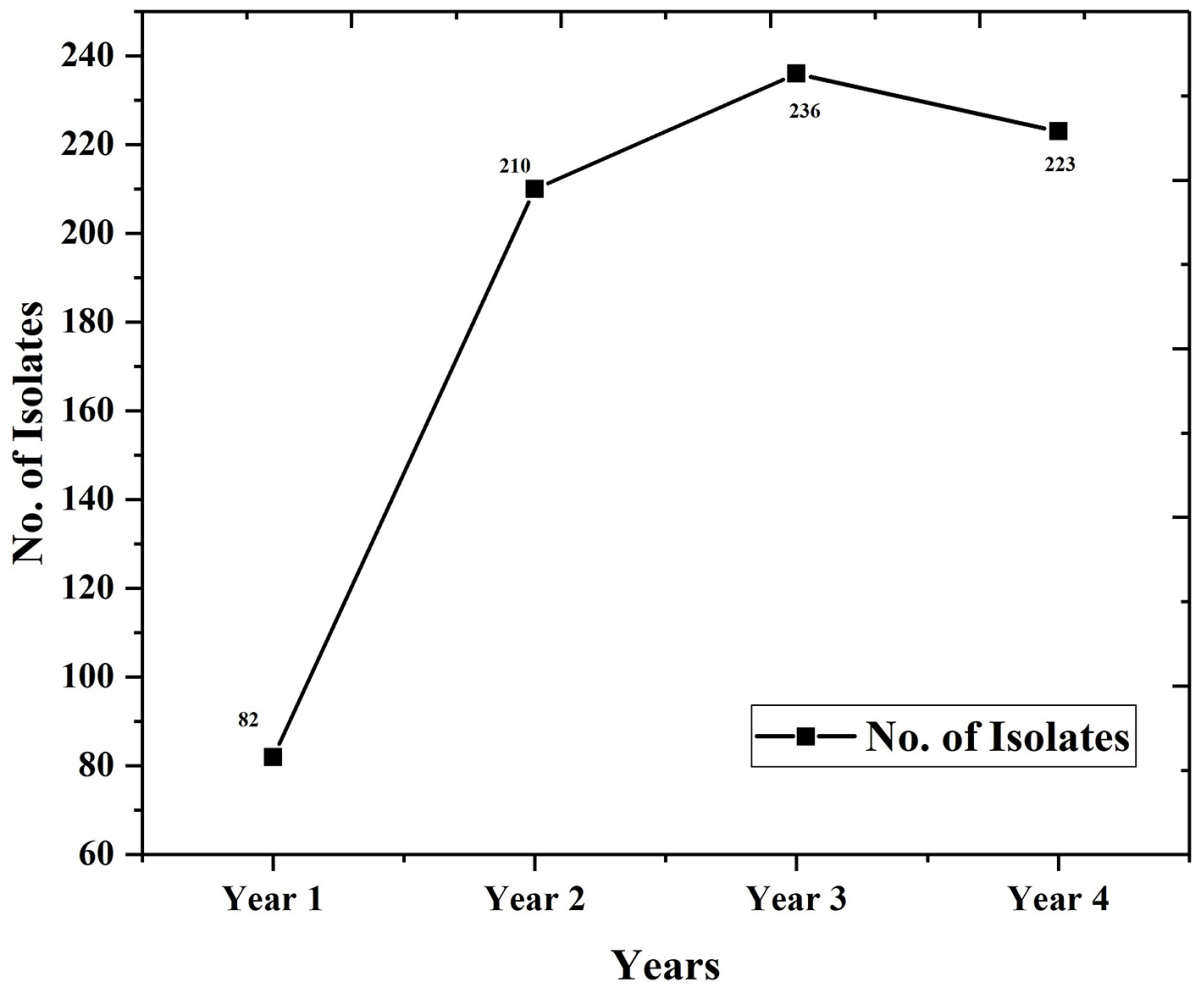
Isolation of *Candida* year wise.

Analysis of the database revealed that 751 *Candida* isolates were recovered from different clinical samples over 4 years. The occurrence level had increased successively over the years except for last year (Figure 1).

The clinical samples processed include sputum, endotracheal (ET) secretions, tracheal aspirates, broncho-alveolar lavages (BAL), urine, stool, vaginal swabs (*VS*), cerebrospinal spinal fluid (CSF), pleural fluid (PF), ascitic fluid (AF), blood, bile, ear, nail, pus from post-surgical wounds, and catheter tips.

### 3.1. *Candida* species isolated from clinical samples

**Figure 2:**
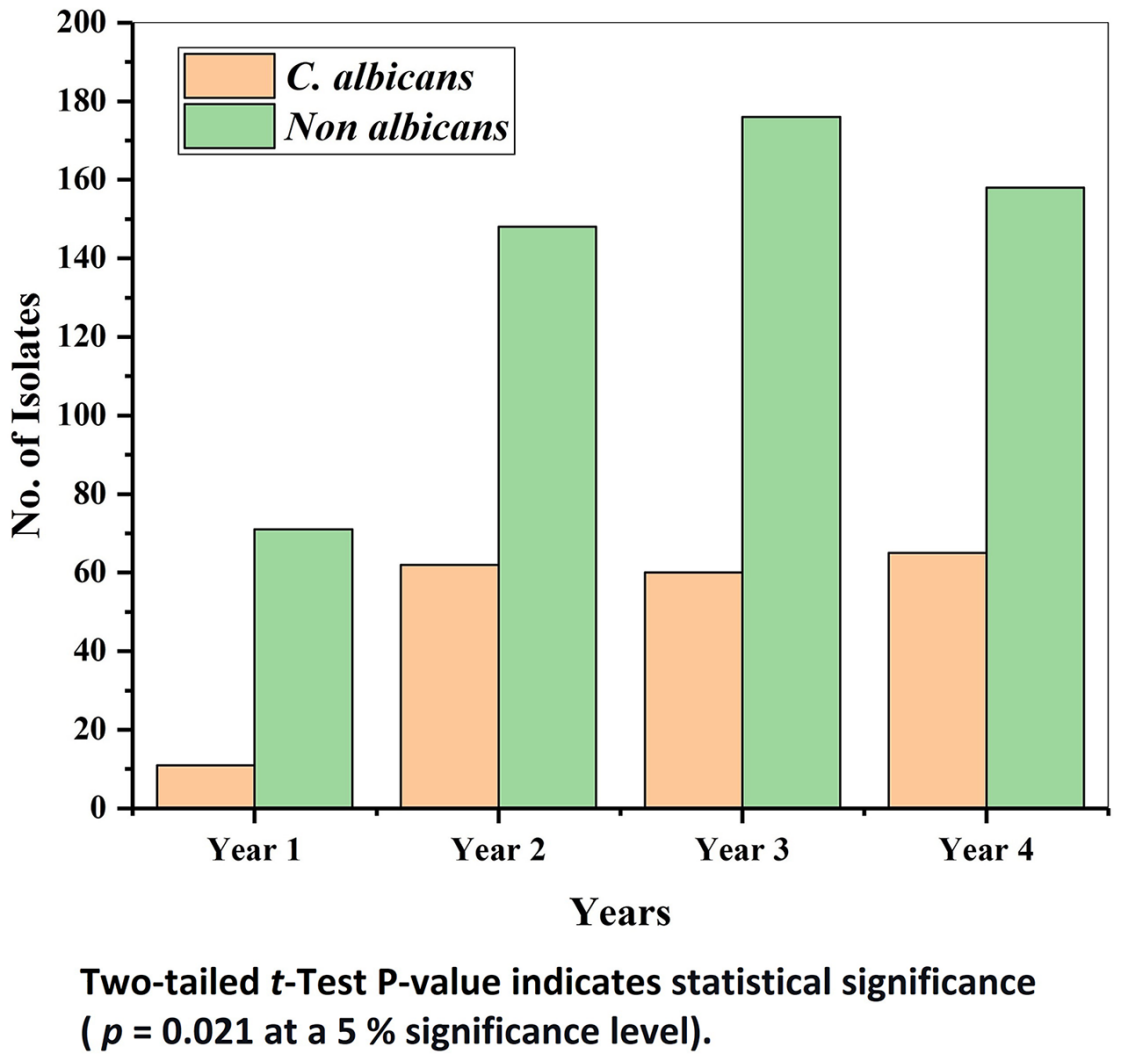
Distribution of *Candida albicans* and non albicans *Candida* species year wise.

Table 1 represents the source of the samples from which *Candida* was isolated during the study period. Of all the samples, urine alone contributed 58.42%, followed by ET (8.92%), pus (8.12%), blood (7.46%), sputum (5.86%), and other samples were < 3%. Table 2 details the distribution of *Candida* species in different clinical samples. It was observed that over the years, there was an increase in the incidence and emergence of different NAC species (Figure 2). A total of 18 different types of *Candida* species were isolated from 751 isolates. NAC (*n* = 553, 73.64%) predominated over *C. albicans* (*n* = 198, 26.36%).

**Table 1:** Isolation of *Candida* year wise in clinical samples (*n* = 751).

Sample	Year 1	Year 2	Year 3	Year 4	Total	%
Urine	58	123	124	133	438	58.32
Sputum	5	13	17	9	44	5.86
Pus	2	16	20	23	61	8.12
Blood	6	6	27	17	56	7.46
Vaginal swab	–	3	9	10	22	2.93
Nail	–	–	1	–	1	0.13
Ear	–	–	1	1	2	0.27
Ascitic fluid	–	1	2	1	4	0.53
Pleural fluid	–	1	1	–	2	0.27
Cerebrospinal fluid	–	1	3	–	4	0.53
Stool	2	1	1	–	4	0.53
Throat swab	–	1	–	–	1	0.13
Bronchoalveolar lavage	–	1	–	–	1	0.13
Fungal culture	1	5	15	11	32	4.26
Endotracheal tube	6	35	11	15	67	8.92
CT	–	3	3	2	8	1.07
Gastric aspirate	1	–	1	1	3	0.40
Bile	1	–	–	–	1	0.13
Total	82	210	236	223	751	
Percent (%)	10.92	27.96	31.42	29.69		

**Table 2:** Distribution of *Candida* species in different clinical samples.

	Urine	Sputum	Pus	Blood	VS	Nail	Ear	AF	PF	CSF	Feces	GA	CT	ET	FC	TS	Bile	Suction tip	Total	%
*C. albicans*	89	20	23	5	14	0	0	1	0	4	1	3	1	18	17	1	0	1	198	26.36
*C. tropicalis*	210	16	20	17	3	1	1	2	2	0	2	0	6	35	6	0	1	0	322	42.88
*C. glabrata*	73	2	4	3	5	0	0	1	0	0	0	0	0	0	0	0	0	0	88	11.72
*C. parapsilosis*	21	1	5	4	0	0	1	0	0	0	0	0	0	3	3	0	0	0	38	5.06
*C. krusei*	5	0	3	9	0	0	0	0	0	0	0	0	0	2	0	0	0	0	19	2.53
*C. haemulonii*	13	1	1	1	0	0	0	0	0	0	0	0	0	1	1	0	0	0	18	2.40
*C. lusitaniae*	5	1	4	0	0	0	0	0	0	0	1	0	0	3	2	0	0	0	16	2.13
*C. guillermondii*	8	0	1	1	0	0	0	0	0	0	0	0	0	4	1	0	0	0	15	2.00
*C. pelliculosa*	0	0	0	8	0	0	0	0	0	0	0	0	0	0	0	0	0	0	8	1.07
*C. famata*	2	2	0	1	0	0	0	0	0	0	0	0	0	1	1	0	0	0	7	0.93
*C. lipolytica*	4	0	0	2	0	0	0	0	0	0	0	0	0	0	0	0	0	0	6	0.80
*C. kefyr*	1	1	0	1	0	0	0	0	0	0	0	0	1	0	0	0	0	0	4	0.53
*C. utilis*	1	0	0	1	0	0	0	0	0	0	0	0	0	0	1	0	0	0	3	0.40
*C. lambica*	1	0	0	1	0	0	0	0	0	0	0	0	0	0	0	0	0	0	2	0.27
*C. norvegensis*	2	0	0	0	0	0	0	0	0	0	0	0	0	0	0	0	0	0	2	0.27
*C. dubliniensis*	1	0	0	1	0	0	0	0	0	0	0	0	0	0	0	0	0	0	2	0.27
*C. rugosa*	1	0	0	1	0	0	0	0	0	0	0	0	0	0	0	0	0	0	2	0.27
*C. colliculosa*	1	0	0	0	0	0	0	0	0	0	0	0	0	0	0	0	0	0	1	0.13
Total	438	44	61	56	22	1	2	4	2	4	4	3	8	67	32	1	1	1	751	100.00
Percent (%)	58.32	5.86	8.12	746	2.93	0.13	0.27	0.53	0.27	0.53	0.53	0.40	1.07	8.92	4.26	0.13	0.13	0.13		

The distribution frequency of *C. albicans over* NAC by the Manne Whitney *U*-test revealed that NAC had the highest mean and sum rank compared to *C. albicans*. The test showed an asymptotic significance (2-tailed) value of *p* and concluded that NAC was statistically significantly higher than the *C. albicans* (*p* = 0.021 at a 5% significance level).

**Figure 3:**
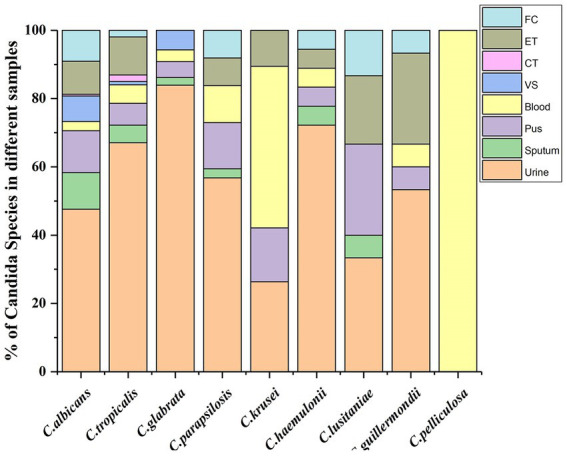
*Candida* species (recovered >1%) from different clinical specimens.

Among NAC, *Candida tropicalis* was the most frequently isolated (*n* = 322, 42.88%) species followed by *Candida glabrata* (*n* = 88, 11.72%), *Candida parapsilosis* (*n* = 38, 5.06%), *Candida krusei* (*n* = 19, 2.53%), *Candida haemulonii* (*n* = 18, 2.4%), *Candida lusitaniae* (*n* = 16, 2.13%), *Candida guillermondii* (*n* = 15, 2.0%). Figure 3 graphically represents the *Candida* species which were recovered at a higher rate (>1%) from different clinical specimens.

Out of 58.32% of Urine samples, *C. tropicalis* accounted to be 210 (46.2%), followed by *C. albicans* 89 (19.58%), *C. glabrata* 73 (16.06%), and *C. parapsilosis* 21 (4.62%). Except for *Candida pelliculosa*, *all* 17 *Candida* were recovered from urine samples. *Candida tropicalis* was isolated from all specimens except CSF, TS, and GA. It was the only species recovered from bile. Apart from *C. lusitaniae*, *Candida norvegensis*, and *Candida colliculosa*, all other species resulted in candidemia. *Candida krusei and C. pelliculosa* were frequently isolated from blood in concurrence with *C. albicans* and *C. tropicalis*; *C. lipolytica* was recovered only from urine and blood, whereas *C. pelliculosa* only from blood. Among 22 vaginal swabs processed, *C. albicans* accounted for 14 (63.64%), followed by *C. glabarata* 5 (22.7%) and *C. tropicalis* 3 (13.6%).

### 3.2. Antifungal susceptibility pattern

**Figure 4:**
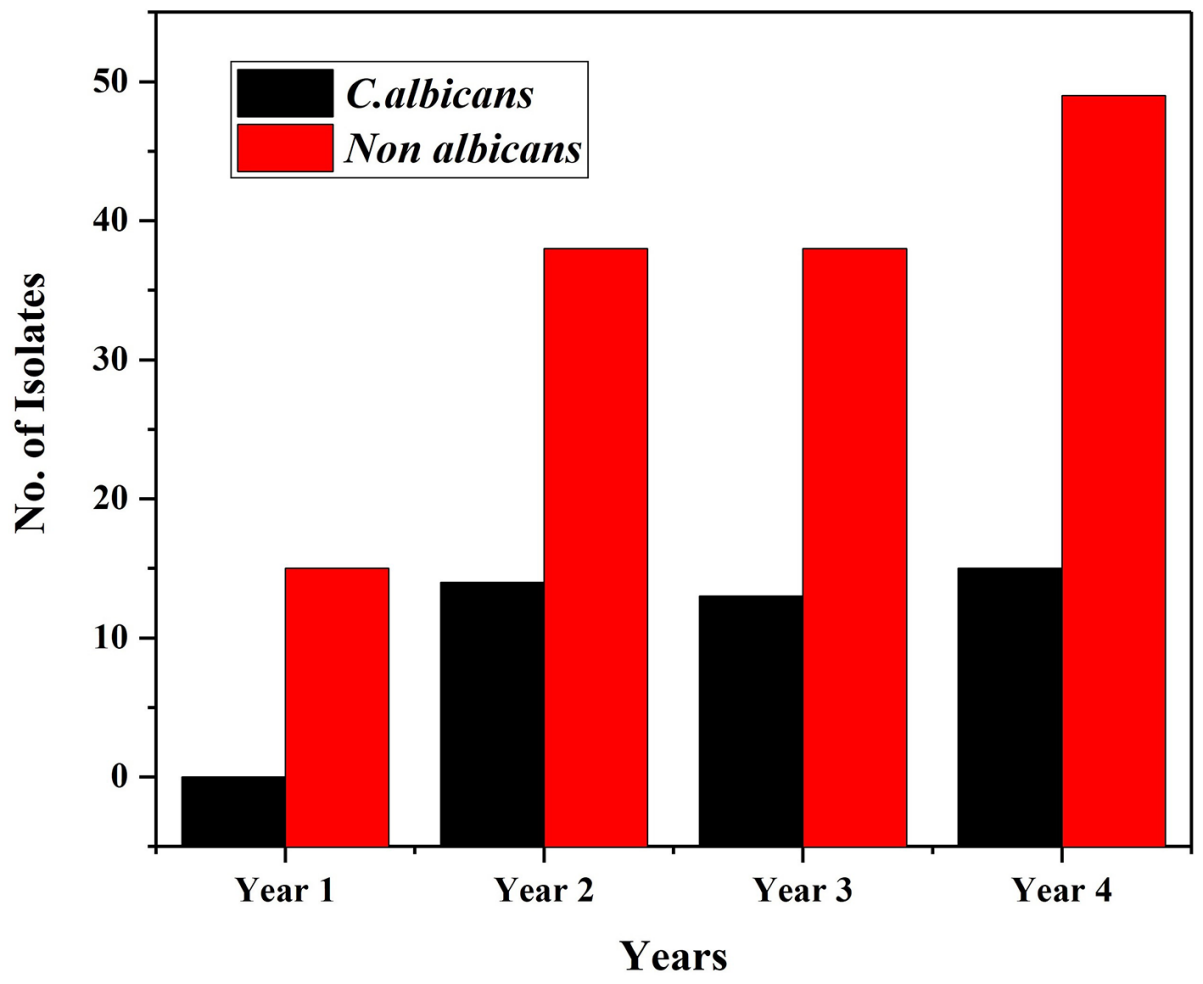
No. of drug resistant isolates of *Candida albicans* and non albicans *Candida year wise*.

**Figure 5:**
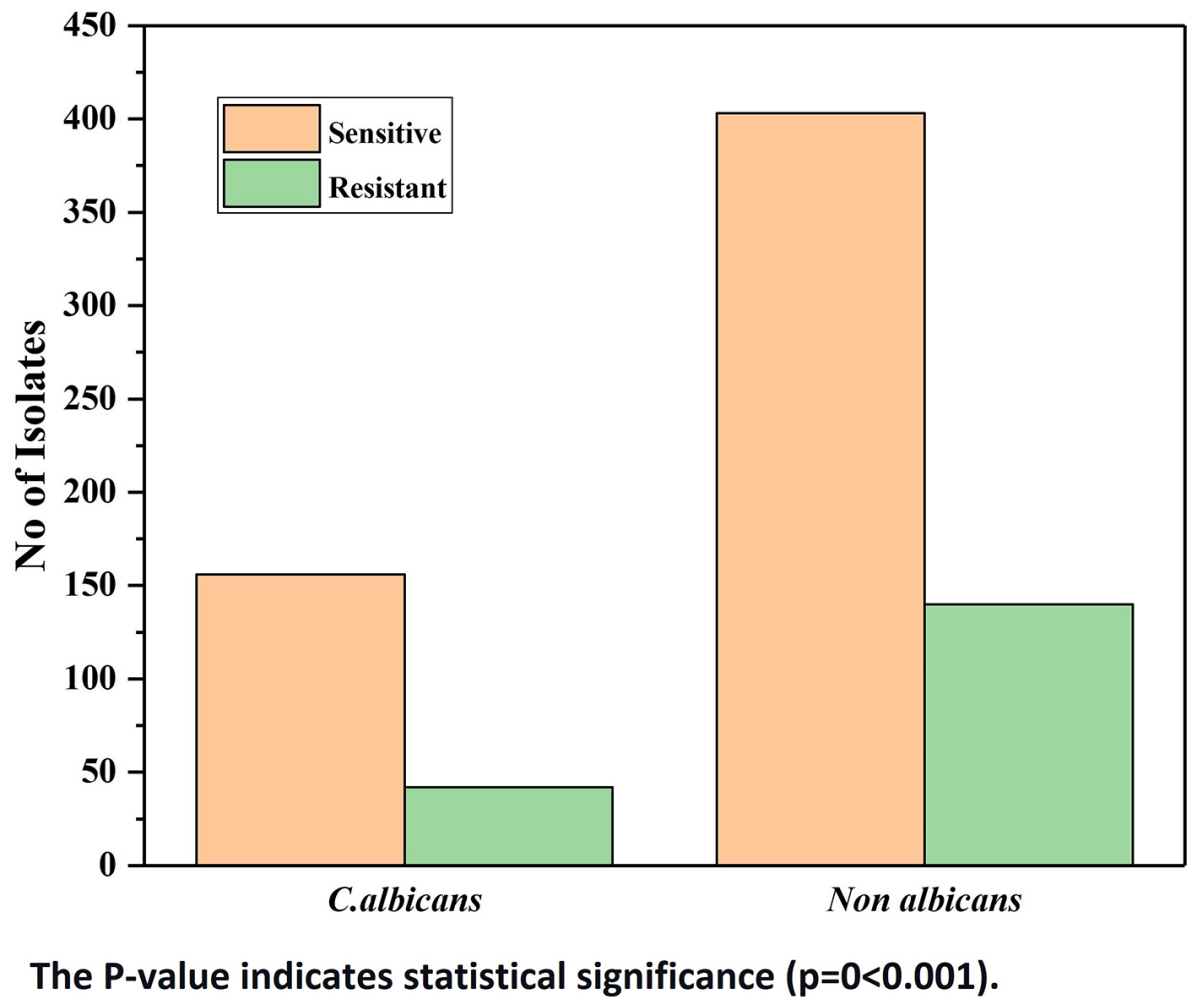
No. of drug resistant isolates of *Candida albicans* and non albicans *Candida*.

Drug susceptibility was carried out for all 751 isolates, but the susceptibility profile was available only for 741 isolates in VITEK with no interpretation for *Candida famata* (7), *Candida lambica* (2) and *C. colliculosa* (1). Among the 741 isolates, 182 (24.56%) showed resistance to one or more drugs tested, and 559 (75.44%) were susceptible to all drugs—Table 3. Both albicans and NAC showed increased resistance to drugs successively. Out of 198\u00B0 *C. albicans*, 156 (78.79%) were sensitive to all antifungals tested, and 42 (21.21%) were resistant to at least one drug—Figures 4, 5. Amongst 553 NAC, 403 (72.88%) were sensitive to all antifungals, and 140 (25.32%) showed resistance to at least one drug. The maximum number of resistant *Candida* species were isolated from urine samples 88/438, followed by ET 24/67, pus 22/61, blood 17/56, and sputum 11/44—Tables 3, 4.

**Table 3:** Resistant *Candidal* isolates from different clinical specimens (*n* = 182).

	Urine	Sputum	Pus	Blood	VS	AF	PF	CSF	Stool	CT	ET	FC	Bile	Total	%
*C. albicans*	22	3	4	0	3	1	0	1	0	0	4	4	0	42	23.08
*C. tropicalis*	33	6	10	4	0	1	1	0	1	3	12	1	1	73	40.11
*C. glabrata*	5	0	1	1	0	1	0	0	0	0	0	0	0	8	4.40
*C. parapsilosis*	4	0	1	2	0	0	0	0	0	0	1	0	0	8	4.40
*C. krusei*	5	0	3	8	0	0	0	0	0	0	2	0	0	18	9.89
*C. haemulonii*	12	1	1	1	0	0	0	0	0	0	1	1	0	17	9.34
*C. lusitaniae*	1	0	1	0	0	0	0	0	0	0	1	1	0	4	2.20
*C. guillermondii*	2	0	1	1	0	0	0	0	0	0	3	0	0	7	3.85
*C. lipolytica*	1	0	0	0	0	0	0	0	0	0	0	0	0	1	0.55
*C. kefyr*	0	1	0	0	0	0	0	0	0	0	0	0	0	1	0.55
*C. norvegensis*	2	0	0	0	0	0	0	0	0	0	0	0	0	2	1.10
*C. rugosa*	1	0	0	0	0	0	0	0	0	0	0	0	0	1	0.55
Total	88	11	22	17	3	3	1	1	1	3	24	7	1	182	
Percent (%)	48.35	6.04	12.09	9.34	1.65	1.65	0.55	0.55	0.55	1.65	13.19	3.85	0.55		

**Table 4:** Antifungal susceptibility profile of *Candida* species.

	Amphotericin B			Flucytosine			Caspofungin			Micafungin			Fluconazole			Voriconazole		
	S	I	R	S	I	R	S	I	R	S	I	R	S	I	R	S	I	R
*C. albicans*	176	12	10	190	0	8	187	5	6	198	0	0	172	11	15	186	0	12
*C. tropicalis*	311	5	6	263	4	55	315	2	5	322	0	0	308	2	12	316	2	4
*C. glabrata*	83	5	0	88	0	0	84	0	4	88	0	0	87	1	0	88	0	0
*C. parapsilosis*	32	2	4	37	0	1	38	0	0	38	0	0	32	2	4	36	2	0
*C. krusei*	16	2	1	13	5	1	8	5	6	19	0	0	15	3	1	18	1	0
*C. haemulonii*	1	2	15	17	1	0	18	0	0	18	0	0	3	14	1	18	0	0
*C. lusitaniae*	15	1	0	13	0	3	16	0	0	16	0	0	16	0	0	16	0	0
*C. guillermondii*	12	1	2	12	0	3	15	0	0	15	0	0	13	2	0	15	0	0
*C. pelliculosa*	8	0	0	8	0	0	8	0	0	8	0	0	8	0	0	8	0	0
*C. lipolytica*	6	0	0	5	1	0	6	0	0	6	0	0	6	0	0	6	0	0
*C. utilis*	3	0	0	3	0	0	3	0	0	3	0	0	3	0	0	3	0	0
*C. kefyr*	4	0	0	3	1	0	4	0	0	4	0	0	4	0	0	4	0	0
*C. norvegensis*	0	1	1	1	1	0	2	0	0	2	0	0	2	0	0	1	0	1
*C. dubliniensis*	0	1	1	2	0	0	2	0	0	2	0	0	2	0	0	2	0	0
*C. rugosa*	0	1	1	2	0	0	2	0	0	2	0	0	2	0	0	2	0	0
Total	667	33	41	657	13	71	708	12	21	741	0	0	673	35	33	719	5	17
Percentage (%)	90.01	4.45	5.53	88.66	1.75	9.58	95.55	1.62	2.83	100.00	0.00	0.00	90.82	4.72	4.45	97.03	0.67	2.29

Of the 182 resistant isolates, 140 (76.92%) NAC and 42 (23.08%) *C. albicans* showed resistance to at least one drug. Among NAC, three species that commonly exhibited resistance were *C. tropicalis* 73 (40.11%), *C. krusei* 18 (9.89%) and *C. haemulonii* 17 (9.34%). The Mann–Whitney *U*-test revealed a significant difference (*p* = 0.028 at 5% significance) in the resistance profile of albicans over NAC.

*In vitro* susceptibility pattern of *Candida* species to six antifungals—amphotericin B, caspofungin, flucytosine, fluconazole, micafungin, and voriconazole has been tabulated in Table 4. For echinocandins such as micafungin-all 741 isolates were susceptible. For caspofungin—maximum resistance was observed by *C. krusei* 6 (31.57%), followed by *C. glabrata* 4 (4.55%), *C. albicans* 6 (3.03%) and *C. tropicalis* 5 (1.55%). For azoles such as voriconazole—12 (6.06%) *C. albicans* and 4 (1.24%) *C. tropicalis* exhibited resistance; for fluconazole—15 (7.58%) *C. albicans*, 12 (3.73%) *C. tropicalis*, 4 (10.52%) *C. parapsilosis*, 1 (5.56%) *C. haemulonii* and 1 (5.2%) *C. krusei* showed resistance. Maximum resistance to flucytosine was observed by *C. tropicalis* 55 (20.91%), *C. albicans* 8 (4.21%), *C. lusitaniae* 3 (23.08%) and *C. guillermondii* 3 (25%). Of all the antifungals tested, *Candida* species were susceptible to micafungin and resistant to flucytosine. Sensitivity to other drugs was in the following order:—voriconazole, caspofungin, fluconazole, and amphotericin B. About 15 (83.3%) of *C. haemulonii* were resistant to amphotericin B. *C. pelliculosa*, *C. utilis*, and *C. dubliniensis* were found sensitive to all drugs.

On comparison of albicans and NAC resistance to all six drugs by the Pearson chi-square test, the value of *p* appeared to be less than 0.05(*p* = 0 < 0.001). Thus, there lies a statistical significance between the organism and the drugs. The drugs and organism association revealed a significant difference at a 5% level (value of *p* 0 < 0.001).

## 4. Discussion

Nosocomial invasive fungal infections are increasing at a higher frequency (20). The present study infers that NAC predominates *C. albicans. C. tropicalis* being the most common species reported, revealed a significant epidemiological shift compared to our previous study from 2010 to 2014 (16). The increased ratio of NAC over *C. albicans* reflected the rise in the incidence of NAC, but the reason for this switchover is unknown. Overall, candiduria was recorded in 58.32%, whereas candidemia was observed in 7.46%. Esmailzadeh et al. (21) and Zakhem et al. (22) reported an increased frequency of NAC compared to *C. albicans* in candiduria and candidemia conditions. Kato et al. (23) and Shenoy et al. (24) reported the prevalence of both albicans and NAC in bile. Among NAC, *C. glabrata* was recorded in both of their studies individually. Studies from Marak (25) and Ballal et al. (26) revealed the presence of only NAC in bile which is in line with our study. In a study by Baghdadi et al. 2016, *C. albicans* was the most common species isolated from endotracheal tubes accounting for 35.7%. Among NAC, *C. glabrata*, *C. parapsilosis*, *C. tropicalis*, and *C. krusei* were recorded (27). A retrospective analysis of epidemiology for candidemia has reported that central venous catheters and endotracheal intubation as the most common risk factors associated with mortality and highlights the significance of considering the possibility of invasive *Candida* infection in patients (28). From vaginal swabs *C. albicans* 63.64% was the predominant species isolated followed by *C. glabrata* and *C. tropicalis*. Studies have shown that elevated levels of estrogen have a direct impact on growth of *Candida* and its adherence to vaginal epithelium suggests an increased incidence of vaginal candidiasis in women. Studying hormone-sensing pathways help in explaining gender bias-associated infections. *C. albicans* is recorded as the predominant colonizer in the female reproductive tract and a major cause of genital thrush (29). The shift in the frequency from albicans to NAC such as *C. glabrata*, *C. parapsilosis*, *C. tropicalis* may be due to increased use of the counter drugs, a short-course of antifungal therapy, and long-term use of azoles (30). NAC has not only overtaken the albicans in uncontrolled distribution but has also evolved in exhibiting drug resistance to azole, polyene and echinocandins drugs which are alarming the situation. Data insights show that *C. tropicalis* parallels *C. albicans* in demonstrating resistance to azole and flucytosine drugs. Increased flucytosine resistance indicates indiscriminate usage of the drug. Apart from *C. albicans*, *NAC such as C. tropicalis*, *C. glabrata*, *and C. krusei* have evolved in exhibiting resistance toward echinocandins. *C. haemulonii* has demonstrated resistance to amphotericin B, which is the mainstay of systemic antifungal therapy (31). For micafungin, all isolates were found to be sensitive.

The limitations of the present study are no other tests were carried out for species confirmation. Also, the data was from a single institution and cannot be extrapolated to other hospitals.

In summary, it is a study which statistically infers the body sites affected by different species of *Candida* and their antifungal resistance patterns. Each species varies in susceptibility level to the distinct group of antifungal agents. The emergence of new species, indiscriminate usage of drugs and rising drug resistance have worsened the situation. Therefore, continuous surveillance, epidemiology, and clinical investigation studies performed locally from each hospital can provide a database to monitor Candidemia infections. The antibiogram profile could guide clinicians to choose appropriate drugs for the emerging *Candida* species and plan effective strategies to control invasive and systemic *Candida* infections.

## Data availability statement

The original contributions presented in the study are included in the article/supplementary material, further inquiries can be directed to the corresponding author.

## Ethics statement

This is an observational study. JSS Academy of Higher Education & Research Ethics Committee has confirmed that no ethical approval is required.

## Author contributions

US and MS contributed to the study’s conception and design. Material preparation, data collection and analysis were performed by US and MS. The first draft of the manuscript was written by US. All authors contributed to the article and approved the submitted version.

## Conflict of interest

The authors declare that the research was conducted in the absence of any commercial or financial relationships that could be construed as a potential conflict of interest.

## Publisher’s note

All claims expressed in this article are solely those of the authors and do not necessarily represent those of their affiliated organizations, or those of the publisher, the editors and the reviewers. Any product that may be evaluated in this article, or claim that may be made by its manufacturer, is not guaranteed or endorsed by the publisher.

## References

[1] Sikora A Zahra F. Nosocomial Infections. [Updated 2023 Jan 23]. In: StatPearls [Internet]. Treasure Island (FL): StatPearls Publishing; 2023 Jan-. Available at: https://www.ncbi.nlm.nih.gov/books/NBK559312/

[2] Badiee P Hashemizadeh Z. Opportunistic invasive fungal infections: diagnosis & clinical management. Indian J Med Res. (2014) 139:195–204. PMID: 24718393 PMC4001330

[3] Viudes A Pemán J Cantón E Úbeda P López-Ribot J Gobernado M. Candidemia at a tertiary-care hospital: epidemiology, treatment, clinical outcome and risk factors for death. Eur J Clin Microbiol Infect Dis. (2002) 21:767–74. doi: 10.1007/s10096-002-0822-1, PMID: 12461585

[4] Pfaller MA Diekema DJ. Epidemiology of invasive candidiasis: a persistent public health problem. Clin Microbiol Rev. (2007) 20:133–63. doi: 10.1128/CMR.00029-06, PMID: 17223626 PMC1797637

[5] Mohandas V Ballal M. Distribution of Candida species in different clinical samples and their virulence: biofilm formation, proteinase and phospholipase production: a study on hospitalized patients in southern India. J Global Infect Dis. (2011) 3:4–8. doi: 10.4103/0974-777X.77288, PMID: 21572601 PMC3068577

[6] Sardi JCO Scorzoni L Bernardi T Fusco-Almeida AM Mendes Giannini MJS. Candida species: current epidemiology, pathogenicity, biofilm formation, natural antifungal products and new therapeutic options. J Med Microbiol. (2013) 62:10–24. doi: 10.1099/jmm.0.045054-0, PMID: 23180477

[7] Abi-Said D Anaissie E Uzun O Raad I Pinzcowski H Vartivarian S. The epidemiology of hematogenous candidiasis caused by different Candida species. Clin Infect Dis. (1997) 24:1122–8. doi: 10.1086/513663, PMID: 9195068

[8] Pappas PG Lionakis MS Arendrup MC Ostrosky-Zeichner L. Kullberg BJ (2018) invasive candidiasis. Nat Rev Dis Primers. (2018) 4:18026. doi: 10.1038/nrdp.2018.26 29749387

[9] Guo LN Yu SY Xiao M Yang CX Bao CM Yu YH. Species distribution and antifungal susceptibility of invasive candidiasis: a 2016-2017 multicenter surveillance study in Beijing. China *Infect Drug Resist*. (2020) 13:2443–52. doi: 10.2147/IDR.S255843, PMID: 32765018 PMC7381087

[10] Guo F Yang Y Kang Y Zang B Cui W Qin B. Invasive candidiasis in intensive care units in China: a multicentre prospective observational study. J Antimicrob Chemother. (2013) 68:1660–8. doi: 10.1093/jac/dkt083, PMID: 23543609

[11] Samra Z Bishara J Ashkenazi S Pitlik S Weinberger M Lapidoth M. Changing distribution of Candida species isolated from sterile and nonsterile sites in Israel. Eur J Clin Microbiol Infect Dis. (2002) 21:542–5. doi: 10.1007/s10096-002-0764-7, PMID: 12172747

[12] Sanglard D Odds FC. Resistance of Candida species to antifungal agents: molecular mechanisms and clinical consequences. Lancet Infect Dis. (2002) 2:73–85. doi: 10.1016/s1473-3099(02)00181-0, PMID: 11901654

[13] Cortegiani A Misseri G Fasciana T Giammanco A Giarratano A Chowdhary A. Epidemiology, clinical characteristics, resistance, and treatment of infections by Candida auris. J Intensive Care. (2018) 6:69. doi: 10.1186/s40560-018-0342-4, PMID: 30397481 PMC6206635

[14] Eggimann P Garbino J Pittet D. 2003. Epidemiology of Candida species infections in critically ill non-immunosuppressed patients. Lancet Infect Dis. (2003) 3:685–702. doi: 10.1016/s1473-3099(03)00801-6, PMID: 14592598

[15] Sanguinetti M Porta R Sali M La Sorda M Pecorini G Fadda G. Evaluation of VITEK 2 and RapID yeast plus systems for yeast species identification: experience at a large clinical microbiology laboratory. J Clin Microbiol. (2007) 45:1343–6. doi: 10.1128/JCM.02469-06, PMID: 17287333 PMC1865843

[16] Shivaswamy U Neelambike SM. A study of candidiasis in HIV reactive patients in a tertiary care hospital, Mysore—South India. Indian J Dermatol Venereol Leprol. (2014) 80:278. doi: 10.4103/0378-6323.132271 24823420

[17] Deorukhkar SC Saini S Mathew S. Non-albicans Candida infection: an emerging threat. Interdiscip Perspect Infect Dis. (2014) 2014:615958. doi: 10.1155/2014/615958, PMID: 25404942 PMC4227454

[18] M27-A3 reference method for broth dilution antifungal susceptibility testing of yeasts; approved standard-third edition (2008) (cited 2020 Dec 4). Available from: www.clsi.org.

[19] Wayne PA. Clinical and laboratory standards institute (CLSI): reference method for broth dilution antifungal susceptibility testing of filamentous fungi (2008) 28(16). Available at: https://clsi.org/media/1897/m27ed4_sample.pdf

[20] Perlroth J Choi B Spellberg B. Nosocomial fungal infections: epidemiology, diagnosis, and treatment. Med Mycol. (2007) 45:321–46. doi: 10.1080/13693780701218689 17510856

[21] Esmailzadeh A Zarrinfar H Fata AM Sen T. High prevalence of Candiduria due to non-Albicans Candida species among diabetic patients: a matter of concern? J Clin Lab Anal. (2018) 32:1–5. doi: 10.1002/jcla.22343 PMC6817075 29076587

[22] Zakhem A Istambouli R Alkozah M Gharamti A Tfaily M Jabbour J. (2021). “Predominance of Candida Glabrata among non-Albicans Candida species in a 16-year study of Candidemia at a tertiary Care Center in Lebanon.” Pathogens 10: 1–10, doi: 10.3390/pathogens10010082 PMC7832319 33477771

[23] Kato H Iizawa Y Nakamura K Gyoten K Hayasaki A Fujii T. The critical role of biliary candidiasis in development of surgical site infections after Pancreatoduodenectomy: results of prospective study using a selective culture medium for Candida species. Biomed Res Int. (2018) 2018:5939724–8. doi: 10.1155/2018/5939724, PMID: 30581862 PMC6276508

[24] Shenoy SM Shalini S Gopal S Tantry B Baliga S Jain A. Clinicomicrobiological analysis of patients with cholangitis. Indian J Med Microbiol. (2014) 32:157–60. doi: 10.4103/0255-0857.129802, PMID: 24713902

[25] Marak MB. Dhanashree B antifungal susceptibility and biofilm production of candida spp. isolated from clinical samples. *Int*. J Microbiol. (2018) 2018:7495218. doi: 10.1155/2018/7495218, PMID: 30405717 PMC6199855

[26] Ballal M Shenoy PA Rodrigues GS Devadas SM Shetty V. Bangera SR biliary tract infections and their microbiological Spectrum-a study from coastal region of southern India. Infection. (2019) 23:253–8. doi: 10.22354/in.v23i3.789

[27] Baghdadi E Khodavaisy S Rezaie S Abolghasem S Kiasat N Salehi Z. Antifungal susceptibility patterns of Candida species recovered from endotracheal tube in an intensive care unit. Adv Med. (2016) 2016:1–6. doi: 10.1155/2016/9242031, PMID: 27642628 PMC5011531

[28] Zhang X-B Yu S-J Yu J-X Gong Y-L Feng W Sun F-J. Retrospective analysis of epidemiology and prognostic factors for candidemia at a hospital in China, 2000-2009. Jpn J Infect Dis. (2012) 65:510–5. doi: 10.7883/yoken.65.510 23183203

[29] Krishnasamy L Krishnakumar S Santharam P Saikumar C. Isolation and identification of Candida species in patients with Vulvovaginal candidiasis. J Pure Appl Microbiol. (2018) 12:2269–73. doi: 10.22207/JPAM.12.4.67

[30] Kumwenda P Cottier F Hendry A Kneafsey D Keevan B Gallagher H. Estrogen promotes innate immune evasion of Candida Albicans through inactivation of the alternative complement system. Cell Rep. (2022) 38:110183. doi: 10.1016/j.celrep.2021.110183 34986357 PMC8755443

[31] Allen U. Antifungal agents for the treatment of systemic fungal infections in children. Can J Infect Dis Med Microbiol. (2010) 21:e116–21. doi: 10.1155/2010/784549, PMID: 22132005 PMC3009580

